# Emergence of Clinically Macrolide-Unresponsive *Mycoplasma pneumoniae* Segmental/Lobar Pneumonia and COVID-19 Pneumonia in Children in Taiwan, 2024–2025

**DOI:** 10.3390/antibiotics15030292

**Published:** 2026-03-13

**Authors:** Hao-Yuan Lee, Chien-Chin Chen, Shu-Hua Ko, Yu-Ling Huang, En-Pen Chang, Cheng-Yi Hsu, Jia Ru Wu, Wei-Hsin Chen, Yu-Chau Hsu, Meng-Yen Li, Yu-Lung Hsu, Wen-Yuan Lee, Chyi-Liang Chen

**Affiliations:** 1 Department of Nursing, Jen-Teh Junior College of Medicine, Nursing and Management, Miaoli 35664, Taiwan; d9700101@gmail.com; 2 Department of Pediatrics, Wei Gong Memorial Hospital, Miaoli 35159, Taiwan; 045198@tool.caaumed.org.tw (C.-C.C.); 045652@tool.caaumed.org.tw (S.-H.K.); 045870@tool.caaumed.org.tw (Y.-L.H.); 045268@tool.caaumed.org.tw (E.-P.C.); 045228@tool.caaumed.org.tw (C.-Y.H.); dreamsykes@hotmail.com (J.R.W.); danger538@gmail.com (W.-H.C.); 010785@tool.caaumed.org.tw (Y.-C.H.); 045441@tool.caaumed.org.tw (M.-Y.L.); 3 School of Medicine, College of Medicine, Fu Jen Catholic University, New Taipei 242062, Taiwan; 4 Division of Pediatric Infectious Diseases, China Medical University Children’s Hospital, China Medical University, Taichung 40447, Taiwan; dan5230@gmail.com; 5 Molecular Infectious Disease Research Center, Chang Gung Memorial Hospital, Taoyuan 333423, Taiwan; 6 Department of Neurosurgery, China Medical University Children’s Hospital, China Medical University, Taichung 40447, Taiwan; 7 Department of Neurosurgery, Wei Gong Memorial Hospital, Miaoli 35159, Taiwan; 8 Department of Microbiology and Immunology, College of Medicine, Chang Gung University, Taoyuan 33302, Taiwan

**Keywords:** macrolide, doxycycline, *Mycoplasma pneumoniae*, COVID-19

## Abstract

**Background:** To date, no study has compared the clinical characteristics of *Mycoplasma pneumoniae*-associated segmental/lobar pneumonia, *Mycoplasma* bronchopneumonia, and COVID-19 pneumonia primarily caused by the NB.1.8.1 variant in children. **Methods**: We examined the epidemiologic trends of pneumonia, segmental/lobar pneumonia, and COVID-19 pneumonia at a teaching hospital from 2015 to 2025. In addition, we compared the clinical characteristics of children hospitalized with *Mycoplasma* segmental/lobar pneumonia, *Mycoplasma* bronchopneumonia, and COVID-19 pneumonia during the NB.1.8.1 variant wave in 2024–2025. **Results**: Between 2015 and 2024, 10,601 pneumonia cases were identified, including 525 cases of segmental/lobar pneumonia and 162 cases of COVID-19 pneumonia. An outbreak of segmental/lobar *M. pneumoniae* pneumonia and COVID-19 pneumonia occurred in Taiwan during 2024–2025. Starting in early 2025, monthly *Mycoplasma* positivity rates among children with segmental/lobar pneumonia and bronchopneumonia exceeded 60%. *Mycoplasma* pneumonia predominantly affected children aged 6–11 years, whereas COVID-19 pneumonia mainly occurred in those younger than 3 years of age. Fever, cough, and rhinorrhea were the most common symptoms in all groups, limiting clinical differentiation. Children with segmental/lobar *Mycoplasma* pneumonia were more likely to present with prolonged fever (>5 days), lymphocytopenia, a neutrophil-to-lymphocyte ratio (NLR) ≥ 3, and elevated C-reactive protein (CRP) levels, each of which was strongly associated with macrolide non-response (all *p* < 0.001). **Conclusions:** Children with segmental/lobar *Mycoplasma* pneumonia demonstrated more severe clinical manifestations. Segmental/lobar involvement and inflammatory markers, such as lymphocytopenia, elevated NLR, and increased CRP levels, were associated with macrolide non-response. These indicators may help guide therapeutic decision-making in pediatric *M. pneumoniae* pneumonia.

## 1. Introduction

Pneumonia is among the most prevalent acute infections and remains the leading cause of death in children globally, responsible for 14% of all deaths in children under five years old [1]. The widespread introduction of pneumococcal conjugate vaccines (PCVs) has substantially altered the epidemiology and etiology of pediatric community-acquired pneumonia (CAP). In Taiwan, the 13-valent pneumococcal conjugate vaccine (PCV13) was introduced into the National Immunization Program (NIP) in 2013 for children aged 2–5 years, and in 2014, the program was extended to include a two-dose schedule for children aged 1–2 years [2]. Following PCV13 implementation, the incidence of invasive pneumococcal disease (IPD) among children under 5 years of age declined markedly, from 18.9 per 100,000 in 2008–2009 to 9.4 in 2013–2014 and further to 6.3 in 2015–2017 [3]. As pneumococcal pneumonia became less prevalent, *Mycoplasma pneumoniae* (MP) emerged as a leading pathogen of pediatric CAP in Taiwan and other regions [4,5].

During the COVID-19 pandemic, Taiwan implemented stringent non-pharmaceutical interventions, including universal masking, contact tracing, quarantine, and social distancing, from May 2020 to April 2023 [6]. These measures substantially reduced the transmission of respiratory pathogens [7], including MP. During this period, pneumonia cases declined sharply; however, a significant resurgence was observed in 2023, followed by an increase in difficult-to-treat segmental or lobar pneumonia cases in 2024, based on our clinical observations. In line with these observations, *M. pneumoniae* infections reemerged worldwide in 2023–2024 following an extended period of unusually low incidence during the COVID-19 pandemic, largely attributed to non-pharmaceutical interventions [8].

The COVID-19 pandemic created a unique context for comparing *Mycoplasma*-associated pneumonia with COVID-19 pneumonia in children. Pediatric COVID-19 pneumonia caused by COVID-19 generally manifests with milder symptoms and less pronounced inflammatory responses, resembling the clinical features observed in other pediatric COVID-19 infections [9,10].

*M. pneumoniae* pneumonia (MPP) is a pneumonia caused by MP [11]. Although MPP is typically self-limiting and associated with a favorable outcome, some cases may progress to severe MPP (SMPP), which can result in extrapulmonary organ involvement as well as systemic complications [12]. Children younger than 5 years of age and those who receive delayed or insufficient antimicrobial therapy are at higher risk for severe MP infection [5].

The rising prevalence of macrolide-resistant *M. pneumoniae* (MRMP), along with co-infections and dysregulated immune responses, has contributed to an increasing number of refractory MPP (RMPP) cases, even after 7 days or more of appropriate macrolide treatment [13,14]. In some children, MPP presenting as lobar pneumonia on imaging may evolve into RMPP, leading to complications such as atelectasis and necrotizing pneumonia [13]. Lobar involvement has been recognized as a risk factor for RMPP and is frequently linked to MRMP infection [15]. In Taiwan, the prevalence of MRMP increased from 12.3–24% before 2017 to 54–88% during 2017–2020 [5,11], reducing the effectiveness of macrolides such as azithromycin and prompting the need for alternative agents, including tetracyclines or fluoroquinolones. A resurgence of MP infections is expected in the post COVID-19 period [16]. Notably, MRMP rates in China remain high, ranging from 50% to 90% among hospitalized patients, with some reports approaching nearly 100% resistance [16,17]. In Taiwan, MP infections typically peak in spring and summer, but incidence declined markedly during the COVID-19 pandemic. Although macrolide resistance reached 85.7% in 2020, it subsequently decreased to 0% during 2022–2023 [11].

The typical radiographic features of MPP include lobar or segmental air-space consolidation, bronchovascular bundle thickening and numerous small centrilobular nodules [18]. In a China study, the annual proportion of segmental or lobar MPP among children with MPP rose markedly during the study period, increasing from 6.4% to 59.6% (*p* < 0.001) [18]. Likewise, in southern Taiwan, lobar pneumonia represented more than 50% of pediatric MPP cases in 2019–2020 [5].

Although polymerase chain reaction (PCR) is regarded as the diagnostic gold standard for MP infection, serological assays—especially IgM testing—are still commonly applied in routine clinical settings because of their availability and practicality [5,19,20]. A previous study in Taiwan reported an initial IgM positivity rate of approximately 63.6% at hospital admission, which increased to 97.5% during follow-up, highlighting the value of paired serologic testing for diagnosis [21].

To date, no study has directly compared the clinical features of *Mycoplasma*-associated segmental/lobar pneumonia, *Mycoplasma* bronchopneumonia, and COVID-19 pneumonia in children. Considering the changing epidemiology of pediatric pneumonia in the post-pandemic era and the differing clinical characteristics of MP-associated segmental/lobar pneumonia and COVID-19 pneumonia, this study aimed to evaluate long-term trends in pediatric pneumonia in Taiwan from 2015 to 2025. We analyzed the epidemiology, clinical features, laboratory results, imaging findings, antimicrobial non-response, and treatment outcomes of *Mycoplasma pneumoniae*-associated pneumonia, particularly segmental/lobar forms and compared them with pediatric COVID-19 pneumonia. By analyzing data collected over a decade, this study seeks to elucidate post-pandemic changes in pneumonia etiology and severity and to provide evidence-based guidance for the diagnosis and management of pediatric respiratory infections in Taiwan.

## 2. Results

### 2.1. Pathology and Epidemiology of Pediatric Pneumonia

**Figure 1:**
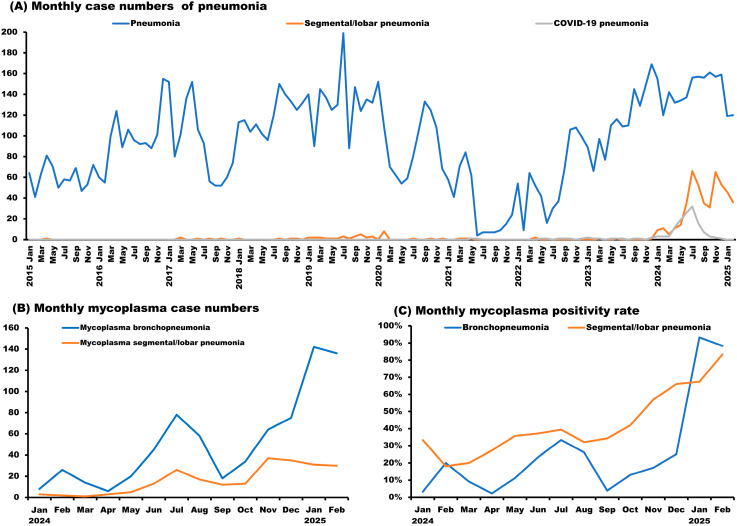
(**A**) Monthly case counts of pediatric pneumonia, segmental/lobar pneumonia, and COVID-19 pneumonia from 2015 to 2025. (**B**) Monthly case counts of pediatric *Mycoplasma pneumoniae* segmental/lobar pneumonia and bronchopneumonia from 2024 to 2025. (**C**) *M. pneumoniae* positivity rates among children with segmental/lobar pneumonia and bronchopneumonia.

Between 2015 and 2024, a total of 10,601 pneumonia cases were identified, including 525 cases of segmental/lobar pneumonia and 162 cases of COVID-19 pneumonia. Notably, 89.5% of segmental/lobar pneumonia cases occurred between January 2024 and February 2025. The highest monthly incidence of segmental/lobar pneumonia was observed in July 2024 (*n* = 66), followed by November 2024 (*n* = 65) (Figure 1A).

Among all pneumonia cases, the highest monthly case count was recorded in July 2019 (*n* = 199), with the second highest in December 2023 (*n* = 169). In contrast, the lowest monthly case count occurred in June 2021 (*n* = 4) (Figure 1A).

### 2.2. Epidemiology of COVID-19 and Mycoplasma Pneumonia in Children

Pediatric COVID-19 pneumonia cases peaked in July 2024 (*n* = 36) (Figure 1A). No cases of COVID-19 related segmental or lobar pneumonia were identified.

Regarding *Mycoplasma* infections, bronchopneumonia cases peaked in January 2025 (*n* = 111), followed by February 2025 (*n* = 106), with the lowest number recorded in April 2024 (*n* = 3). Segmental/lobar pneumonia due to *Mycoplasma* reached its highest monthly incidence in November 2024 (*n* = 37), followed by December 2024 (*n* = 35), and was least frequent in March 2024 (*n* = 1) (Figure 1B).

Among children with bronchopneumonia, the highest monthly *Mycoplasma* positivity rate was observed in January 2025 (93.3%), followed by February 2025 (88.3%), and the lowest in April 2024 (2.3%). In children with segmental/lobar pneumonia, the highest positivity rate occurred in February 2025 (83.3%), followed by January 2025 (37.4%), with the lowest rate in February 2024 (18.2%) (Figure 1C).

### 2.3. Sex and Age Distribution in Children with Mycoplasma vs. COVID-19 Pneumonia

Between 2024 and 2025, a significantly higher proportion of males was observed among children with COVID-19 bronchopneumonia (66.4%) compared with those diagnosed with *Mycoplasma* segmental/lobar pneumonia or bronchopneumonia (both ≤56.1%; *p* = 0.049) (Table 1). The median age of children with *Mycoplasma* segmental/lobar pneumonia or bronchopneumonia was 8 years, which was significantly older than that of children with COVID-19 bronchopneumonia (2 years).

Infants younger than 1 year constituted a larger percentage of admissions for COVID-19 bronchopneumonia than for *Mycoplasma* segmental/lobar pneumonia or bronchopneumonia (11.2% vs. both ≤9.27%; *p* < 0.001). Likewise, over 67% of children with COVID-19 bronchopneumonia were under 3 years of age, whereas less than 20% of those with *Mycoplasma* segmental/lobar pneumonia or bronchopneumonia were in this age group (*p* < 0.001). Conversely, children aged 6–11 years represented more than half of the *Mycoplasma* segmental/lobar pneumonia or bronchopneumonia cases but accounted for only 2.1% of COVID-19 bronchopneumonia cases (*p* < 0.001) (Table 1).

### 2.4. Symptom Comparison Between Mycoplasma and COVID-19 Pneumonia in Children

Children with COVID-19 bronchopneumonia and those with *Mycoplasma* segmental/lobar pneumonia showed a significantly higher rate of fever (both >90%) compared with children with *Mycoplasma* bronchopneumonia (64.1%; *p* < 0.001). In addition, high-grade fever (>40.0 °C or >39.4 °C) was observed more frequently in children with COVID-19 bronchopneumonia than in those with *Mycoplasma* segmental/lobar pneumonia or bronchopneumonia (both *p* ≤ 0.001) (Table 1).

Fever lasting ≥3 days was more common in children with *Mycoplasma* segmental/lobar pneumonia or bronchopneumonia (both >79%) than in those with COVID-19 bronchopneumonia (42.6%; *p* < 0.001). Fever duration of ≥5 days occurred more frequently in *Mycoplasma* segmental/lobar pneumonia (48.2%) than in *Mycoplasma* bronchopneumonia (25.0%), and both were significantly higher than in COVID-19 bronchopneumonia (1.4%; *p* < 0.001). Similarly, fever lasting ≥7 days was observed more often in *Mycoplasma* segmental/lobar pneumonia (32.5%) than in *Mycoplasma* bronchopneumonia (10.3%) or COVID-19 bronchopneumonia (0.7%; *p* < 0.001).

Cough was reported in all children with *Mycoplasma* segmental/lobar pneumonia or bronchopneumonia (100%), a significantly greater proportion than that observed in children with COVID-19 bronchopneumonia (88.8%; *p* < 0.001). Rhinorrhea was the third most common symptom across all groups (65–76%; *p* = 0.077).

Gastrointestinal symptoms were more prevalent in children with *Mycoplasma* segmental/lobar pneumonia or bronchopneumonia, including vomiting (7–11% vs. 1.4%) and abdominal pain (9–12% vs. 1.4%), compared with children with COVID-19 bronchopneumonia (*p* = 0.002) (Table 1).

### 2.5. Laboratory Comparison Between Mycoplasma and COVID-19 in Children

Among laboratory parameters, lymphocytopenia, elevated neutrophil-to-lymphocyte ratio (NLR), and increased C-reactive protein (CRP) were more strongly associated with *Mycoplasma* segmental/lobar pneumonia than with *Mycoplasma* bronchopneumonia or COVID-19 bronchopneumonia (all *p* < 0.001) (Table 2).

Lymphocytopenia (<2000/μL) was most common in *Mycoplasma* segmental/lobar pneumonia (60.5%), followed by *Mycoplasma* bronchopneumonia (48.6%), and least frequent in COVID-19 bronchopneumonia (21.7%) (*p* < 0.001). Similar patterns were observed at stricter cutoffs: <1500/μL (33.8%, 19.6%, and 10.5%, respectively) and <1000/μL (7.9%, 4.8%, and 0.7%, respectively; all *p* < 0.001).

NLR ≥3 was most prevalent in *Mycoplasma* segmental/lobar pneumonia (53.9%), followed by *Mycoplasma* bronchopneumonia (41.1%) and COVID-19 bronchopneumonia (21.7%) (*p* < 0.001). Likewise, NLR ≥5 was more frequent in *Mycoplasma* segmental/lobar pneumonia (31.1%) than in *Mycoplasma* bronchopneumonia (16.5%) or COVID-19 bronchopneumonia (18.9%) (*p* < 0.001).

Elevated CRP levels were also most pronounced in *Mycoplasma* segmental/lobar pneumonia. CRP >1 mg/dL was observed in 81.1% of these cases, compared with 60.7% in *Mycoplasma* bronchopneumonia and 20.3% in COVID-19 bronchopneumonia cases (*p* < 0.001). CRP >3 mg/dL occurred in 32.4%, 14.7%, and 0.7%, respectively (*p* < 0.001), with a similar trend for CRP >2 mg/dL (Table 2).

In contrast, leukocytosis (>12,000/μL) was more frequently observed in *Mycoplasma* bronchopneumonia and COVID-19 bronchopneumonia (≥44%) than in *Mycoplasma* segmental/lobar pneumonia (*p* < 0.001). Neutrophilia (>7500/μL) was also more common in *Mycoplasma* bronchopneumonia (>45%) than in the other two groups (*p* < 0.001).

Anemia was significantly more prevalent in *Mycoplasma* segmental/lobar pneumonia (11.8%) compared with *Mycoplasma* bronchopneumonia and COVID-19 bronchopneumonia (both <1%; *p* < 0.001). Thrombocytopenia (<200,000/μL) followed a similar pattern (12.7%, 9.5%, and 1.4%, respectively; *p* < 0.001). Elevated liver enzymes (AST > 38 U/L or ALT > 44 U/L) were more common in *Mycoplasma* bronchopneumonia (9.5%) than in *Mycoplasma* segmental/lobar pneumonia or COVID-19 bronchopneumonia (both <2%; *p* < 0.001).

### 2.6. Treatment Analysis

Macrolide non-response—defined as persistent fever and no clinical or radiographic improvement 3 days after treatment initiation—was significantly more common in children with *Mycoplasma* segmental/lobar pneumonia compared to those with *Mycoplasma* bronchopneumonia (82.0% vs. 41.7%; *p* < 0.001) (Table 2). Consequently, doxycycline was prescribed more frequently for segmental/lobar pneumonia (82.0% vs. 41.7%; *p* < 0.001).

Among the 724 children with *Mycoplasma* pneumonia, macrolide non-response was significantly associated with lymphocyte counts < 2000/μL, NLR ≥ 3, CRP > 2 mg/dL, radiographic segmental/lobar involvement, and fever duration ≥5 days (all *p* < 0.001) (Table 3).

## 3. Discussion

To our knowledge, this study is the first comprehensive analysis comparing the clinical presentations of children hospitalized with *Mycoplasma*-associated segmental/lobar pneumonia, *Mycoplasma* bronchopneumonia, and COVID-19 pneumonia during the 2024–2025 outbreak in Taiwan. It also represents the first report of pediatric pneumonia cases predominantly caused by the Omicron NB.1.8.1 variant in 2024. Additionally, this study identifies useful biomarkers, including the NLR, lymphocyte count, and CRP level, which may help clinicians select appropriate antibiotic therapy for children with *M. pneumoniae* infection in the setting of high macrolide non-response.

Globally, MP infections rebounded in 2023–2024 after a prolonged period of unusually low incidence due to COVID-19 related non-pharmaceutical interventions [22,23,24,25], a pattern also observed in our study. The highest monthly case numbers for overall pneumonia, segmental/lobar pneumonia, and COVID-19 pneumonia were all recorded in 2024, following the pediatric COVID-19 outbreak in 2022–2023. In contrast, the lowest monthly case count was observed in June 2021 (*n* = 4), reflecting the effect of Taiwan’s strict preventive measures from May 2020 to April 2023, including contact tracing, universal masking, and social distancing, which markedly suppressed the occurrence of pediatric pneumonia [6,10].

An increasing trend in both *Mycoplasma* bronchopneumonia and segmental/lobar pneumonia was found in both positivity rates and case numbers, which has been newly identified worldwide. The *Mycoplasma* bronchopneumonia positivity rate revealed an increased trend from January 2024 (3.2%) to January 2025 (93.3%). The *Mycoplasma* segmental/lobar pneumonia positivity rate also revealed an increasing trend from February 2024 (18.2%) to February 2025 (83.3%). The *Mycoplasma* bronchopneumonia monthly case number revealed an increasing trend from January 2024 (*n* = 5) to January 2025 (*n* = 111). The *Mycoplasma* segmental/lobar pneumonia monthly case number revealed an increasing trend from March 2024 (*n* = 1) to November 2024 (*n* = 37).

Pediatric COVID-19 pneumonia cases appeared and reached their peak in July 2024 (*n* = 36), primarily caused by the SARS-CoV-2 variant NB.1.8.1 [26].

A male predominance (>50%) was observed among children with *Mycoplasma* pneumonia in our study, aligning with data from a Chinese study conducted between 2000 and 2009 [18] but differing from findings in a Korean study from 2006 to 2007 [27]. Furthermore, a higher proportion of males (>65%) was noted among children with COVID-19 pneumonia in our cohort, consistent with our earlier report on pediatric COVID-19 infections in 2024 [10].

The median age of children with *Mycoplasma* pneumonia in this study was 8 years, including those with segmental/lobar pneumonia and bronchopneumonia. This finding is comparable to a study conducted in China between 2000 and 2009 [18] but higher than that reported in a Korean study from 2006 to 2007 [27]. In contrast, the median age of children with COVID-19 pneumonia in our cohort was 2 years, which is similar to our previous report on pediatric COVID-19 infections from 2022 to 2024 [10]. The median age of children with *Mycoplasma* pneumonia (8 years) was significantly higher than that of children with COVID-19 pneumonia (2 years) in our study (*p* < 0.001). Most cases of *Mycoplasma* pneumonia occurred in children aged 6–11 years, whereas COVID-19 pneumonia predominantly affected those younger than 3 years. This distinct age distribution may assist in differentiating infections caused by these two pathogens.

In our study, children with COVID-19 pneumonia were more likely to present with a peak temperature >40 °C or >39.4 °C compared with those with *Mycoplasma* pneumonia. In our previous study, more than 50% of children with influenza A had a peak temperature >39.4 °C [10], which was higher than that observed in children with either *Mycoplasma* or COVID-19 pneumonia in the present study. Additionally, the proportion of children with COVID-19 pneumonia who had a peak temperature >39.4 °C (45.5%) in this study was higher than that reported for children with COVID-19 infections (26–40%) in our previous study [10].

In our study, children with *Mycoplasma* segmental/lobar pneumonia had higher proportions of prolonged fever (≥3, ≥5, and ≥7 days) compared with those with *Mycoplasma* bronchopneumonia, and both groups had longer fever duration than those with COVID-19 pneumonia. In a study conducted in China between 2000 and 2009 [18], the mean duration of fever in children with *Mycoplasma* segmental/lobar pneumonia (4.13 days) was longer than that in children with *Mycoplasma* bronchopneumonia (3.02 days), which is consistent with our findings.

Distinguishing *Mycoplasma* pneumonia from COVID-19 pneumonia in pediatric patients based solely on clinical manifestations is difficult. In our cohort, fever (>64%), cough (>88%), and rhinorrhea (>65%) were the most frequently reported symptoms in both conditions. These symptoms were also commonly documented in children with influenza or COVID-19 infections in our previous study [10], highlighting their nonspecific nature.

A study conducted at a teaching hospital in China between January and November 2023 reported that cough (99.4%) and fever (98.8%) were the predominant symptoms in children with *M. pneumoniae* infection, whereas sore throat was less common (3.2%) [24]. Likewise, a Taiwanese study of community-acquired *Mycoplasma* pneumonia in 2010 found that fever (99.2%) and cough (96.9%) were the leading symptoms, followed by tachypnea (22.8%) [28]. Collectively, these findings emphasize that clinical symptoms alone provide limited discriminatory value when differentiating *Mycoplasma* pneumonia from other respiratory tract infections.

In this study, children with *Mycoplasma* segmental/lobar pneumonia or bronchopneumonia in this study exhibited higher rates of gastrointestinal symptoms, such as vomiting, diarrhea, and abdominal pain, compared with those with COVID-19 pneumonia (*p* ≤ 0.042). Similar findings were reported in a previous Taiwanese study of children with community-acquired MP in 2010, where vomiting occurred in 20.5%, abdominal pain in 15.7%, and diarrhea in 11.8% of cases [28].

In a Korean study conducted between 2006 and 2007 [27], the mean lymphocyte count (/μL) was higher in children with *Mycoplasma* bronchopneumonia (2700) than in those with segmental/lobar *Mycoplasma* pneumonia (1900). Similarly, in our study, lymphocytopenia, defined as lymphocyte counts <2000/μL, <1500/μL, or <1000/μL, was more frequently observed in children with segmental/lobar *Mycoplasma* pneumonia than in those with *Mycoplasma* bronchopneumonia, and it was also more common than in children with COVID-19 pneumonia (*p* < 0.005).

In this 2015–2025 pediatric cohort, children with MP pneumonia who exhibited peripheral lymphocytopenia demonstrated distinct clinical and laboratory characteristics compared with those without lymphopenia. These findings are consistent with previous studies linking lymphocyte depletion to more severe MPP, including prolonged fever, greater radiographic consolidation (segmental/lobar involvement), and refractory clinical courses [29]. Lymphopenia, defined as a reduction in the absolute lymphocyte count or lymphocyte proportion in peripheral blood, likely reflects immune dysregulation in the host response to MP infection and has consistently been associated with increased disease severity.

Retrospective clinical studies have reported that pediatric patients with MPP accompanied by lymphocytopenia are more likely to have prolonged fever, extended hospital stays, and an increased incidence of segmental or lobar consolidation, indicating that reduced lymphocyte counts are associated with more severe lung involvement [29]. Notably, segmental/lobar radiographic patterns, such as those observed in our cohort, have been linked to decreased absolute lymphocyte counts and amplified inflammatory responses, emphasizing the clinical relevance of lymphocyte measurements as prognostic indicators [29].

Additionally, prospective investigations have demonstrated that specific lymphocyte subsets, including CD3^+^ T lymphocytes and CD3^+^CD8^+^ cytotoxic T cells, are significantly diminished in children with severe or refractory MPP compared with those with milder disease [30,31]. This reduction in lymphocyte subsets likely reflects compromised adaptive immune function, which may impair microbial clearance and contribute to disease progression. Our findings, showing lower lymphocyte counts in children with severe segmental/lobar MP, are consistent with these observations and further underscore the importance of routine lymphocyte evaluation in assessing disease severity.

In addition to absolute lymphocyte counts, inflammatory indices such as the NLR have been explored as prognostic markers in MPP. An elevated NLR at admission has been linked to adverse outcomes, including necrotizing pneumonia and refractory MPP in children, and has been associated with prolonged fever, higher rates of intensive care unit admission, and greater healthcare utilization [32]. In our cohort, NLR ≥3 or ≥5 was significantly more frequent in children with *Mycoplasma* segmental/lobar pneumonia than in those with *Mycoplasma* bronchopneumonia (both *p* < 0.001).

Elevated CRP levels (>1 mg/dL or >3 mg/dL) were more frequently observed in children with *Mycoplasma* segmental/lobar pneumonia than in those with *Mycoplasma* bronchopneumonia and were also higher than in children with COVID-19 pneumonia (both *p* < 0.001). In a Chinese study conducted between 2000 and 2009, the proportion of cases with abnormal CRP levels was greater in children with segmental/lobar pneumonia (30.9%) than in those with bronchopneumonia (23.7%) [18], which is consistent with our findings. Similarly, a Korean study from 2006 to 2007 reported higher mean CRP levels in children with segmental/lobar pneumonia (5.1 mg/dL) compared with bronchopneumonia (2.1 mg/dL) [27]. Elevated AST (>38 U/L) and ALT (>44 U/L) were detected in 9.5% of children with *Mycoplasma* bronchopneumonia, a higher proportion than in those with segmental/lobar pneumonia or COVID-19 pneumonia.

In a Korean study from 2006–2007 [27], the mean leukocyte count (×10^3^/μL) was higher in children with *Mycoplasma* bronchopneumonia (8.7) compared with those with segmental/lobar pneumonia (7.4). Similarly, in our study, leukocytosis (WBC > 12,000/μL) was observed in a greater proportion of children with *Mycoplasma* bronchopneumonia (45%) than in those with segmental/lobar pneumonia (3.9%). The higher prevalence of leukocytosis in children with non-segmental/lobar MP may reflect differences in host immune response and disease distribution.

MP infection triggers immune-mediated inflammation, often resulting in systemic inflammatory markers and leukocyte activation, particularly in earlier or less consolidated disease. In contrast, segmental/lobar pneumonia represents a more localized inflammatory process within the lung parenchyma, which may be associated with lower peripheral white blood cell and lymphocyte counts despite more pronounced radiographic consolidation. These observations suggest that systemic leukocytosis is more prominent in non- segmental/lobar or early inflammatory phenotypes, whereas localized lobar involvement may not provoke the same degree of peripheral leukocyte elevation [27].

According to World Health Organization hemoglobin criteria, anemia was identified in 11.8% of children with *Mycoplasma* segmental/lobar pneumonia but was rare in those with *Mycoplasma* bronchopneumonia or COVID-19 pneumonia (both <1%). Thrombocytopenia (platelet count < 200,000/μL) was observed in approximately 10% of children with *Mycoplasma* segmental/lobar pneumonia or bronchopneumonia, whereas it was uncommon among children with COVID-19 pneumonia (1.4%).

Non-response to macrolide therapy and increased doxycycline use were more common in children with segmental/lobar *Mycoplasma* pneumonia (82%) than in those with bronchopneumonia (41.7%). In Taiwan, *M. pneumoniae* accounts for approximately 15% of pediatric community-acquired pneumonia (CAP), with a reported macrolide resistance rate of 23% [33]. Furthermore, the average proportion of macrolide-resistant MPP (MR-MPP) was 54.3% among children with PCR-confirmed MPP enrolled between July 2016 and June 2019 [34]. In Taiwan, MRMP prevalence increased from 12.3–24% before 2017 to 54–88% during 2017–2020 [5,11]. Although macrolide resistance peaked at 85.7% in 2020, it declined to 0% during 2022–2023 [11] but increased to 54.4% in 2024–2025 according to our study.

Taken together, these findings suggest that peripheral lymphopenia may serve not only as an indicator but also as a potential contributor to severe MPP. Decreased lymphocyte counts and impaired T cell-mediated immunity may result from immune exhaustion or apoptosis triggered by excessive inflammatory responses, thereby weakening the host’s ability to suppress *M. pneumoniae* proliferation and eliminate infection. In clinical practice, routine evaluation of lymphocyte counts, NLR, and other inflammatory indices—together with radiographic evidence such as segmental/lobar consolidation—may improve early risk stratification and guide decisions regarding antibiotic escalation and supportive management.

Several limitations should be considered in both our study and the existing literature. First, the retrospective design conducted at a single center may restrict the broader applicability of our findings. Second, detailed immune profiling—including lymphocyte subset analysis and functional assessments such as T cell proliferation and cytokine profiling—was not performed, particularly in a prospective pediatric setting.

Future multicenter studies incorporating comprehensive immune characterization and longitudinal follow-up are needed to clarify the causal relationship between biomarkers (e.g., lymphopenia, NLR, and CRP) and the severity of MPP. Such investigations may also help determine whether targeted immunomodulatory therapies can improve outcomes and reduce the risk of severe disease.

## 4. Materials and Methods

### 4.1. Study Design

We examined the epidemiology of pediatric patients diagnosed with pneumonia, segmental/lobar pneumonia, and COVID-19 pneumonia at Wei Gong Memorial Hospital (WGMH), an 828-bed hospital affiliated with the China Medical University Strategic Alliance in northern Taiwan, from January 2015 to February 2025. Pneumonia cases were identified using inpatient diagnostic codes—ICD-9-CM codes (480.x–486) prior to 2015 and ICD-10-CM codes (J12.xx–J18.x) thereafter—and diagnoses were confirmed and categorized as segmental/lobar pneumonia or bronchopneumonia based on chest radiograph interpretations by radiologists. COVID-19 pneumonia was defined as radiographically confirmed pneumonia in children with a positive COVID-19 PCR or rapid antigen test.

To reduce potential bias associated with healthcare accessibility or socioeconomic disparities, we excluded children without National Health Insurance coverage. Furthermore, we conducted a detailed analysis of the clinical characteristics of children hospitalized with *M. pneumoniae* segmental/lobar pneumonia, *M. pneumoniae* bronchopneumonia, and COVID-19 pneumonia from January 2024 to February 2025.

### 4.2. Radiographic Definitions

Chest radiographs were independently reviewed by two pediatricians who were blinded to the patients’ clinical information. Pneumonia was defined radiographically as the presence of pulmonary consolidation on plain chest radiographs, including either segmental/lobar pneumonia, characterized by homogeneous consolidation involving a single lobe or lung segment, or bronchopneumonia, characterized by patchy, peribronchial, or multifocal areas of consolidation affecting one or more lobes, with or without visible air bronchograms or pleural effusion.

Two pediatricians, one of whom was a pediatric infectious disease specialist, interpreted the chest radiographs independently, and the diagnosis of CAP was confirmed when their interpretations were concordant.

### 4.3. Laboratory Confirmation of Mycoplasma pneumoniae Infections

*M. pneumoniae* infection was defined by meeting at least one of the following criteria: (1) first-time positivity for *M. pneumoniae*-specific IgM at admission or during acute-stage follow-up, with cases showing prior IgM positivity excluded; (2) a ≥4-fold rise in *Mycoplasma*-specific IgG antibody titers between the acute and convalescent phases. The exclusion criteria were as follows: (1) co-infection with other pathogens; (2) prior IgM positivity; (3) incomplete hospitalization records.

Single-target molecular PCR testing for *M. pneumoniae* was not performed at our hospital, primarily because it is not reimbursed by Taiwan’s National Health Insurance (NHI), whereas *M. pneumoniae*-specific IgM and IgG tests are covered. Two self-paid FilmArray respiratory panel tests were performed but excluded from analysis because additional pathogens were detected in these cases.

Previous studies in Taiwan have reported an initial IgM positivity rate of approximately 63.6% at admission, increasing to 97.5% during follow-up, supporting the diagnostic value of paired serologic testing [21].

After excluding 89 children with prior IgM positivity, other detected pathogens, or incomplete hospitalization records, 724 children with *M. pneumoniae* infection were included. Of these, 708 had first-time IgM positivity at admission or during acute follow-up, whereas the remaining 16, who lacked initial IgM positivity, showed a ≥4-fold increase in IgG titers between the acute and convalescent stages.

For qualitative detection of IgM and semi-quantitative measurement of IgG antibodies against MP in human serum or plasma, the LIAISON^®^ *M. pneumoniae* IgM and IgG assays (Biotrin International Ltd., Dublin, Ireland) were performed using a chemiluminescence immunoassay (CLIA) on the LIAISON analyzer [35]. Specific IgM and IgG antibodies were measured using indirect sandwich CLIA methods. For IgM detection, magnetic particles coated with *M. pneumoniae* lysate enriched with recombinant antigens were used to enhance specificity and sensitivity. The samples, calibrators, and controls were incubated in a buffer containing goat anti-human IgG to reduce interference from IgG antibodies and rheumatoid factor. Bound IgM antibodies were detected using an isoluminol-labeled mouse monoclonal anti-human IgM antibody.

For IgG detection, magnetic particles were coated with recombinant *M. pneumoniae* antigens, and bound IgG antibodies were detected using an isoluminol-labeled mouse monoclonal anti-human IgG antibody. After each incubation, unbound material was removed by washing. Chemiluminescence was initiated by the addition of starter reagents, and emitted light was measured as relative light units (RLUs) using a photomultiplier, indicating the presence of *M. pneumoniae*-specific IgM or IgG antibodies.

### 4.4. Laboratory Confirmation of COVID-19 Infections

A diagnosis of COVID-19 infections was established based on a positive result from either the Xpert^®^ Xpress CoV-2 Plus reverse transcription polymerase chain reaction (RT-PCR) assay (Sunnyvale, CA, USA) or the Panbio™ COVID-19 rapid antigen test. The exclusion criteria were the following: (1) co-infection with other pathogens; (2) incomplete hospitalization records. Nasopharyngeal swab specimens were analyzed using the GeneXpert^®^ Dx system (Sunnyvale, CA, USA), which incorporates internal controls to ensure assay accuracy [36,37]. Detection of COVID-19 RNA was performed with the Xpert^®^ Xpress CoV-2 Plus assay, targeting the E, N, and RdRP genes, with respective limits of detection of 200, 403, and 70 copies/mL.

The Panbio™ COVID-19 rapid antigen test (Abbott, Wiesbaden, Germany) was conducted using nasal or oral specimens. The test strip contains monoclonal anti-chicken IgY at the control line and immobilized anti-SARS-CoV-2 antibodies at the test line. A positive result was indicated by the appearance of a visible test line, while assay validity was confirmed by a clear control line [38].

### 4.5. Clinical Definitions

Anemia was defined according to the World Health Organization (WHO) age-specific criteria [39]. Pneumonia was diagnosed based on the WHO clinical case definition, including symptoms such as cough or difficulty breathing—and confirmed using WHO radiological criteria.

Treatment response was defined as defervescence, with body temperature dropping below 38.0 °C and maintained for ≥24 h within 3 days of initiating therapy, along with no progression of pneumonic lesions on chest radiographs [40]. Non-response to macrolide therapy in children with *M. pneumoniae* infection was defined as persistent fever or worsening of pneumonic lesions on radiographs after 3 days of initial macrolide treatment.

### 4.6. Ethical Statements and Informed Consent Statement

This study was approved by the Institutional Review Board of China Medical University Hospital, Taiwan (IRB No. CMUH114-REC1-075). Informed consent was waived because of the retrospective nature of the study, and the databases used contained only de-identified data.

### 4.7. Statistical Analysis

Pearson’s chi-squared test or Fisher’s exact test, as appropriate, was used to compare categorical variables. Continuous variables were analyzed using linear regression, one-way ANOVA, or the Wilcoxon rank-sum test, as appropriate. All statistical analyses were two-sided, and significance was defined as *p* < 0.05. The Kolmogorov–Smirnov test for larger samples (≥50) and the Shapiro–Wilk test for smaller samples (<50) were used to assess normality of continuous data. In both tests, a *p*-value > 0.05 indicates that the null hypothesis cannot be rejected, suggesting the data follow a normal distribution [41].

## 5. Conclusions

During 2024–2025, Taiwan experienced an outbreak of segmental/lobar MP and COVID-19 pneumonia. Beginning in early 2025, monthly *Mycoplasma* positivity rates exceeded 60% among children with both segmental/lobar pneumonia and bronchopneumonia. *Mycoplasma*-associated segmental/lobar pneumonia and bronchopneumonia primarily affected children aged 6–11 years, whereas COVID-19 pneumonia was most common in those under 3 years of age. Fever, cough, and rhinorrhea were the most common symptoms in both infections, making clinical differentiation challenging. Approximately half of the children with segmental/lobar MP had fever lasting more than 5 days, a higher proportion than that observed in *Mycoplasma* bronchopneumonia or COVID-19 pneumonia. Segmental/lobar MP was also associated with lymphocytopenia, a NLR ≥ 3, and elevated CRP levels, all of which were strongly linked to macrolide non-response, with rates up to 82%. Among children with MP, macrolide non-response was significantly associated with lymphocyte counts < 2000/μL, NLR ≥ 3, CRP > 2 mg/dL, segmental/lobar involvement, and fever duration ≥5 days. These clinical and laboratory indicators may help guide treatment decisions in pediatric MP.

## Figures and Tables

**Table 1:** Distribution of sex, age, and clinical presentations in children with Mycoplasma segmental/lobar pneumonia, Mycoplasma bronchopneumonia, and COVID-19 pneumonia.

Characteristics	*Mycoplasma*			COVID-19 Bronchopneumonia (N = 143)	*p*-Value ^3^
	Segmental/Lobar Pneumonia (N = 228)	Bronchopneumonia (N = 496)	*p*-Value ^2^		
Male gender, *n* (%)	128 (56.1) ^1^	273 (55.0)	0.782	95 (66.4)	0.049
Age	8 (5–11)	8 (2–13)	0.634	2 (2–3)	<0.001
Age < 1 years	1 (0.4)	46 (9.27)	<0.001	16 (11.2)	<0.001
Age < 3 years	18 (7.9)	97 (19.5)	<0.001	97 (67.8)	<0.001
Age 3–5 years	45 (19.7)	147 (29.6)	0.005	41 (28.6)	0.018
Age 6–11 years	127 (55.7)	249 (50.2)	0.169	3 (2.1)	<0.001
Age 12–18 years	38 (16.7)	3 (0.6)	<0.001	2 (1.4)	<0.001
Fever	210 (92.1)	318 (64.1)	<0.001	141 (98.6)	<0.001
Highest temperature > 40 °C	45 (19.7)	95 (19.1)	0.854	48 (33.6)	0.001
Highest temperature > 39.4 °C	148 (36.2)	150 (30.1)	<0.001	65 (45.5)	<0.001
Duration of fever ≥ 5 days	110 (48.2)	124 (25.0)	<0.001	2 (1.4)	<0.001
Cough	228 (100)	496 (100)	1.000	127 (88.8)	<0.001
Rhinorrhea	164 (71.9)	373 (75.2)	0.350	94 (65.7)	0.077
Sore throat	9 (3.95)	26 (5.2)	0.532	32 (22.3)	<0.001
Vomiting	18 (7.9)	51 (10.3)	0.342	2 (1.4)	0.003
Diarrhea	10 (4.4)	28 (5.6)	0.401	1 (0.7)	0.042
Headache	9 (3.9)	23 (4.6)	0.846	1 (0.7)	0.095
Abdominal pain	21 (9.2)	55 (11.1)	0.444	2 (1.4)	0.002

^1^ Data are presented as number (%) or median (interquartile range, 25th–75th percentile). ^2^ *p*-values in the fourth column were calculated for comparisons between two groups: children with *Mycoplasma* segmental/lobar pneumonia and children with *Mycoplasma* bronchopneumonia. ^3^ *p*-values in the rightmost column were calculated for comparisons among three groups: children with *Mycoplasma* segmental/lobar pneumonia, children with *Mycoplasma* bronchopneumonia, and children with COVID-19 pneumonia.

**Table 2:** Laboratory findings and treatments in children with *Mycoplasma* segmental/lobar pneumonia, *Mycoplasma* bronchopneumonia, and COVID-19 pneumonia.

Characteristics	*Mycoplasma*			COVID-19 Bronchopneumonia (N = 143)	*p*-Value ^3^
	Segmental/Lobar Pneumonia (N = 228)	Bronchopneumonia (N = 496)	*p*-Value ^2^		
Lymphocyte < 2000/μL	138 (60.5) ^1^	241 (48.6)	0.003	31 (21.7)	<0.001
Neutrophil-to-lymphocyte ratio ≥ 3	123 (53.9)	204 (41.1)	0.001	31 (21.7)	<0.001
CRP > 2 mg/dL	119 (52.2)	149 (30.0)	<0.001	1 (0.7)	<0.001
WBC > 12,000/µL	9 (3.9)	223 (45.0)	<0.001	64 (44.8)	<0.001
WBC < 4000/µL	1 (0.9)	2 (0.4)	1.000	0 (0)	0.741
Neutrophil > 7500/µL	36 (15.8)	224 (45.2)	<0.001	32 (22.4)	<0.001
Neutrophil < 1500/μL	1 (0.9)	2 (0.4)	1.000	0 (0)	0.741
WHO-defined anemia	27 (11.8)	1 (0.2)	<0.001	1 (0.7)	<0.001
Platelet < 200,000/μL	29 (12.7)	47 (9.5)	0.186	2 (1.4)	0.001
AST > 38 U/L	1 (0.4)	47 (9.5)	<0.001	2 (1.4)	<0.001
ALT > 44 U/L	1 (0.4)	47 (9.5)	<0.001	2 (1.4)	<0.001
Non-response to macrolide therapy	187 (82.0)	207 (41.7)	<0.001	NA ^4^	NA
Doxycycline usage	187 (82.0)	207 (41.7)	<0.001	NA	NA

^1^ Data are presented as number (%) or median (interquartile range, 25th–75th percentile). ^2^ *p*-values in the fourth column were calculated for comparisons between two groups: children with *Mycoplasma* segmental/lobar pneumonia and children with *Mycoplasma* bronchopneumonia. ^3^ *p*-values in the rightmost column were calculated for comparisons among three groups: children with *Mycoplasma* segmental/lobar pneumonia, children with *Mycoplasma* bronchopneumonia, and children with COVID-19 pneumonia. ^4^ NA: Not applicable (antibiotics are not indicated for viral infections).

**Table 3:** Comparison of clinical characteristics in children with Mycoplasma pneumonia: responders versus non-responders to macrolide therapy.

Characteristics	Non-Responders to Macrolide (N = 394)	Responders to Macrolide (N = 330)	*p*-Value
Lymphocyte < 2000/μL	277 (70.3) ^1^	108 (32.7)	<0.001
Neutrophil-to-lymphocyte ratio (NLR) ≥ 3	221 (56.1)	103 (31.2)	<0.001
CRP > 2 mg/dL	218 (55.3)	98 (29.7)	<0.001
Radiological segmental/lobar involvement	187 (47.5)	41 (12.4)	<0.001
Duration of fever ≥ 5 days	207 (52.5)	111 (33.6)	<0.001

^1^ Data are presented as number (%) or median (interquartile range, 25th–75th percentile).

## Data Availability

The original contributions presented in this study are included in the article, and further inquiries can be directed to the corresponding author.
